# Equine Rhinitis A Virus and Its Low pH Empty Particle: Clues Towards an Aphthovirus Entry Mechanism?

**DOI:** 10.1371/journal.ppat.1000620

**Published:** 2009-10-09

**Authors:** Tobias J. Tuthill, Karl Harlos, Thomas S. Walter, Nick J. Knowles, Elisabetta Groppelli, David J. Rowlands, David I. Stuart, Elizabeth E. Fry

**Affiliations:** 1 Institute of Molecular and Cellular Biology and Astbury Centre for Structural Molecular Biology, Faculty of Biological Sciences, University of Leeds, Leeds, United Kingdom; 2 Division of Structural Biology, University of Oxford and Oxford Protein Production Facility, The Henry Wellcome Building for Genomic Medicine, Headington, Oxford, United Kingdom; 3 Institute for Animal Health, Pirbright Laboratory, Pirbright, Surrey, United Kingdom; Stanford University School of Medicine, United States of America

## Abstract

Equine rhinitis A virus (ERAV) is closely related to foot-and-mouth disease virus (FMDV), belonging to the genus *Aphthovirus* of the *Picornaviridae*. How picornaviruses introduce their RNA genome into the cytoplasm of the host cell to initiate replication is unclear since they have no lipid envelope to facilitate fusion with cellular membranes. It has been thought that the dissociation of the FMDV particle into pentameric subunits at acidic pH is the mechanism for genome release during cell entry, but this raises the problem of how transfer across the endosome membrane of the genome might be facilitated. In contrast, most other picornaviruses form ‘altered’ particle intermediates (not reported for aphthoviruses) thought to induce membrane pores through which the genome can be transferred. Here we show that ERAV, like FMDV, dissociates into pentamers at mildly acidic pH but demonstrate that dissociation is preceded by the transient formation of empty 80S particles which have released their genome and may represent novel biologically relevant intermediates in the aphthovirus cell entry process. The crystal structures of the native ERAV virus and a low pH form have been determined via highly efficient crystallization and data collection strategies, required due to low virus yields. ERAV is closely similar to FMDV for VP2, VP3 and part of VP4 but VP1 diverges, to give a particle with a pitted surface, as seen in cardioviruses. The low pH particle has internal structure consistent with it representing a pre-dissociation cell entry intermediate. These results suggest a unified mechanism of picornavirus cell entry.

## Author Summary

Picornaviruses are small animal viruses comprising an RNA genome protected by a roughly spherical protein shell with icosahedral symmetry. How the RNA is introduced into the cytoplasm of the host cell to initiate replication is unclear since they have no lipid envelope to facilitate fusion with cellular membranes. Instead, they become internalized into endocytic vesicles whence the viral genome must be delivered through the vesicle membrane, into the cytoplasm. In some picornaviruses (enteroviruses), genome delivery is proposed to be coordinated by an intact particle inducing pore formation in the membrane through which the genome can be transferred directly without exposure to the hostile vesicle environment. In contrast, other picornaviruses (aphthoviruses e.g. ERAV, FMDV) present a dilemma by appearing to simply fall apart in acidified vesicles. Here we show that acid treatment results in the formation of an intact but transient aphthovirus empty particle from which the genome has been released. We have determined the crystal structures of the ERAV particle at native and acidic pH. The acid induced structure is consistent with a destabilized particle en-route to disassembly. We propose that the entry process for this group of viruses involves externalisation of the RNA from a novel capsid intermediate and unifies in principle the entry process for all picornaviruses.

## Introduction

The *Picornaviridae* is a family of small non-enveloped RNA viruses, classified into several genera including *Enterovirus* (*e.g.* poliovirus, PV; human rhinovirus, HRV), *Aphthovirus* (*e.g.* foot-and-mouth disease virus) and *Cardiovirus* (*e.g.* Mengovirus). Equine rhinitis A virus (ERAV) shares physicochemical properties such as buoyant density, base composition and acid lability with foot-and-mouth-disease virus (FMDV) [1],[2]. The nucleotide sequence of ERAV also links it most closely to FMDV [3]–[5] and ERAV is now included alongside FMDVs in the aphthovirus genus of the *Picornaviridae* [6].

The clinical disease caused by ERAV most closely resembles the febrile respiratory tract infections attributable to rhinoviruses rather than the systemic disease observed in entero- and cardiovirus infections [4], [7]–[9]. However, broad host cell range, viraemia and persistent infection are associated with ERAV; features not seen with rhinovirus infections but reminiscent of the consequences of FMDV infection [1]. ERAV also shares unusual features of genome structure and organization with FMDV [4]. For example, it encodes two species of L protein and possesses a 16 amino acid 2A protein. However, a further unusual feature of the FMDV genome, the three copies of 3B (VPg), is not present in ERAV. The similarities between the two viruses suggest that ERAV may be a useful model system for analyzing the biology of FMDV.

**Figure 1:**
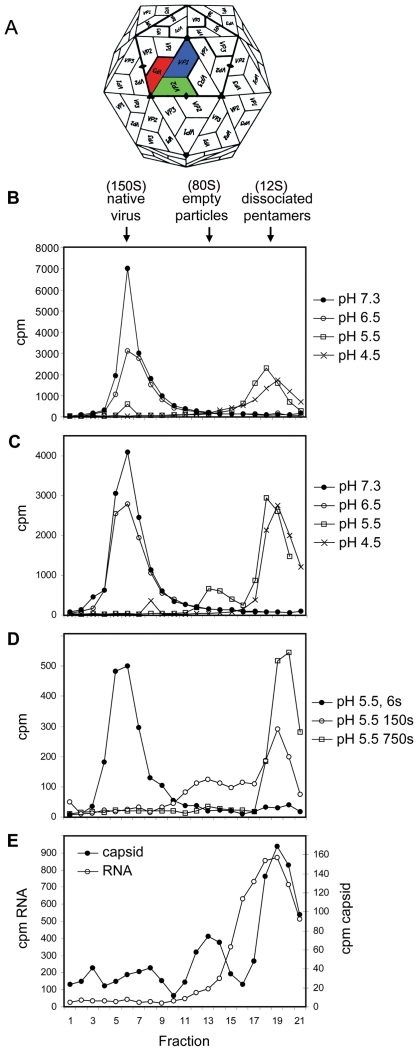
ERAV dissociates at low pH via a transient empty particle. (A) Schematic depiction of a picornaviral icosahedral capsid (diameter approximately 300 Å) showing the pseudo T = 3 arrangement of 60 copies of each of the viral proteins VP1-4. An individual subunit of the icosahedron (the biological protomer deriving from the uncleaved polyprotein) is coloured conventionally: VP1 blue, VP2 green, VP3 red. VP4 is not shown as it is internal. (B) Sedimentation (from right to left) of radiolabelled virus and products of low pH induced dissociation after exposure to solutions with pH 7.3, 6.5, 5.5 or 4.5 as indicated. Arrows indicate positions on the gradient of virus, empty particles and dissociated pentameric subunits. (C) Sedimentation as (B) after exposure to solutions also containing BSA. (D) Sedimentation of radiolabelled virus and products of low pH induced dissociation after exposure to pH 5.5 for 6, 150 or 750 seconds, as indicated. (E) Empty capsids and RNA sediment separately after exposure to pH 5.5.

The ∼300 Å diameter non-enveloped picornavirus capsid encloses a single-stranded RNA genome coding for a poly-protein which is post-translationally cleaved by viral proteases to yield the structural and non-structural viral proteins. The mature capsid comprises 60 copies of each of four proteins: VP1-4 (with molecular weights of 25, 25, 22 and 11 kDa respectively for ERAV) [2]. VP1-3 are composed of eight-stranded β-sandwiches, with strands denoted CHEF and BIDG respectively on the two sheets, which form a pseudo T = 3 icosahedral lattice (Figure 1A) [10] with the CHEF sheet exposed on the capsid surface and BIDG to the interior. VP4, and the N-termini of VP1 and VP3, are located internally.

Crystallographic structures are available for representative viruses from several genera of the *Picornaviridae* [11]. These share, within a genus, on average 86% sequence identity, with VP1 being the most variable protein. Although there are underlying structural similarities between all picornaviruses, they cluster into two groups which correlate with their mechanisms of uncoating. The enterovirus capsid proteins possess several extended surface loops giving rise to a circular cleft (canyon) around the icosahedral five-fold axes, which often functions as a site for receptor attachment [12],[13]. Receptor binding frequently destabilizes the virion and triggers the uncoating process, which proceeds via altered (A) 135S particles in which VP4 is partially or completely absent and the VP1 N-terminus is externalized, ultimately producing icosahedral 80S empty particles which have expelled the genomic RNA [14]. The A particle exposes hydrophobic sequences which are thought to facilitate membrane attachment and transport of the viral RNA through the membrane into the cytoplasm. In contrast cardio- and aphthoviruses lack the circular cleft, have weaker inter-pentamer interactions and dissociate directly to pentamers at low pH with no evidence for receptor-binding induced conformational changes [15]. These distinct pathways for genome-release would appear to have profound consequences for the mechanisms by which the viral RNA is delivered to the cytoplasm to initiate infection. The enteroviruses might introduce their RNA into the cytoplasm by forming a pore in the membrane through which the genome is transferred directly from the capsid, or by disrupting the vesicle membrane prior to release of the genome within the cytoplasm. Both of these scenarios could protect the RNA from exposure to the potentially damaging environment of the lumen of the entry vesicle. In contrast it is difficult to envisage how RNA transfer can safely occur for viruses, such as the FMDVs, that appear to simply dissociate into RNA and protein subunits under the influence of reduced pH in the endosome.

To investigate how similar ERAV and FMDV are in terms of structure and to probe further the cell entry mechanism for aphthoviruses we have determined crystal structures of ERAV at two different pHs, using highly optimised methods since the yield of purified virus was very low. These structures confirm that ERAV is most closely similar to FMDV. In addition the structure of a low-pH form shows internal changes consistent with a pre-dissociation state and biochemical analyses show that ERAV dissociates in acid conditions into pentameric subunits via a transient 80S intermediate particle which has lost the genomic RNA. These results suggest that there may be more similarities in the entry mechanisms for different picornaviruses than has been thought.

## Results

### ERAV dissociates to pentameric capsid subunits at low pH

FMDV dissociates in mildly acidic conditions into pentameric capsid subunits presumed critical for the release of genomic RNA from the particle during the process of cell entry. For ERAV it is known that virus infectivity is sensitive to low pH [2], but the effect of acidification on the integrity of the particle has not been reported. We therefore investigated the effects of exposing radiolabelled ERAV particles to low pH by analyzing their sedimentation in sucrose gradients (Figure 1B). At pH 7.3 or 6.5, particles sedimented at approximately 150S, the expected position for native virus. However, after exposure to pH 5.5, only a minor peak of native virus was seen and the majority of the radioactivity sedimented more slowly, consistent with the dissociation of particles into 12S pentameric capsid subunits. After exposure to pH 4.5, a similar profile was seen, but with no signal for native virus.

### Dissociation is preceded by the ‘transient’ formation of an intact empty particle

Radiolabelled ERAV was exposed to low pH conditions as above but with the addition of 0.1 mg/ml bovine serum albumin (BSA) and 2 mM CaCl_2_, conditions which stabilise uncoating intermediates of poliovirus [16]. At pH 5.5, a minor peak was detected in the expected position for a 75–80S particle (Figure 1C), potentially equivalent to the 80S empty particles produced during uncoating of poliovirus (PV) and human rhinovirus (HRV). To investigate the kinetics of formation of this particle (hereafter termed 80S), radiolabelled virus was diluted 20-fold into pH 5.5 buffer and re-adjusted to neutral pH (by addition of Tris pH 7.5 to 500 mM) after various exposure times (Figure 1D). Brief exposure (6 s) of ERAV to pH 5.5 had no effect on virus sedimentation. However, after exposure for 150 s, a mixture of 80S and dissociated pentamers was observed while after 750 s only pentamers were detectable. To confirm that the 80S particle was empty of RNA, virus preparations with ^35^S labelled capsids or ^3^H labelled RNA were mixed and exposed to pH 5.5. Sedimentation of these samples (Figure 1E) showed a minor protein peak at 80S from the capsid, while no equivalent signal was detected for the RNA, consistent with the formation of an empty particle. Together these data demonstrate that viral RNA is lost from an intact but transient ERAV empty particle, before the dissociation to pentameric subunits.

### Determination of crystal structures

**Figure 2:**
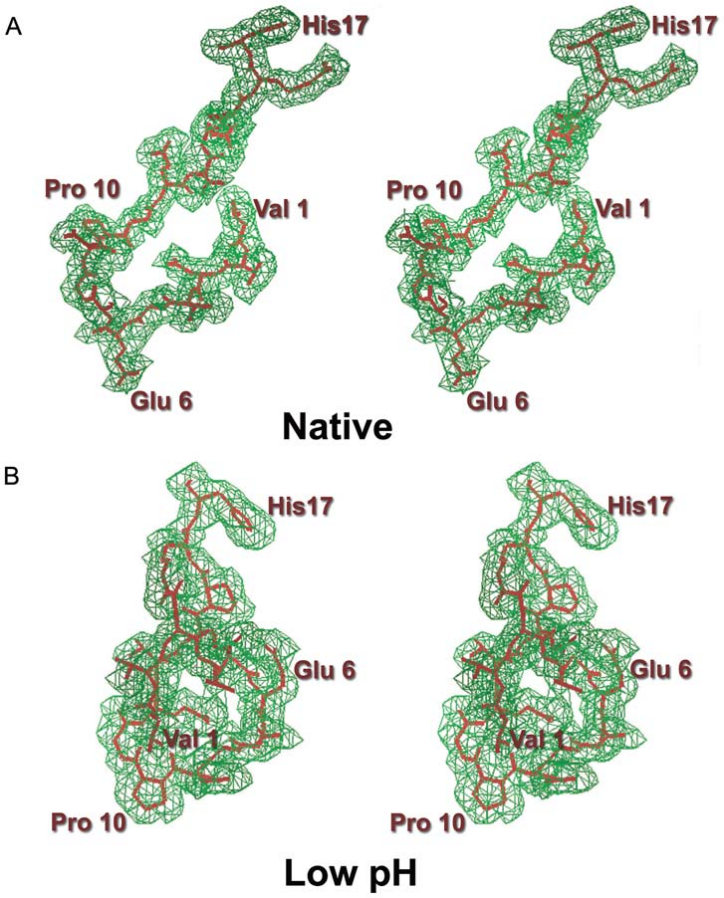
Electron density. (A) Stereo view of the 2Fo-Fc averaged electron density map (green) and coordinates (red) for the N-terminal 17 residues of VP1 in the low pH ERAV particle. (B) Stereo view of the 2Fo-Fc averaged electron density map (green) and coordinates (red) for the N-terminal 17 residues of VP1 in the native ERAV particle. The direction of view is the same as for (A) – note the dramatic rearrangement of this portion of VP1. Both maps are contoured at approximately 0.8 σ.

Micro-crystallization facilities and the microdiffractometer-equipped station BM14 at the ESRF allowed data collection from small crystals grown from limited supplies of virus (Table 1 and data not shown). A particle data set, and a receptor soaked data set (Fry et al, in preparation) were collected from crystals grown at pH 4.6, from approximately 11 µl of 2.7 mg/ml virus solution. With thirty-fold non-crystallographic redundancy, the electron density map of the low pH particle at 3.0 Å resolution clearly differentiated residues that differ between ERAV and FMDV A10_61_ [17] despite incomplete (41%) sampling of the reciprocal lattice. The model built into this map (Figure 2A) comprised residues 1–246 of VP1, 31–230 of VP2, 1–226 of VP3 and 16–36 of VP4 (residues 1–30 of VP2, 1–15 and 37–80 of VP4 were too flexible to be reliably modelled – a difference map confirmed that these segments were not truncated by the averaging envelope). N-terminal sequencing of VP2 verified that the VP0 cleavage occurs at the homologous position to FMDV with no evidence for an upstream cleavage [18]. The final R factor for the model was 28.3% with 98.1% of the residues having allowed Ramachandran angles [19].

**Table 1:** X-ray data collection and structure refinement statistics.

Data collection/processing	Low pH	Native
Space group	C222_1_	P2_1_
Unit cell (Å)	a = 344.8, b = 531.4 c = 488.3	a = 314.0, b = 497.8, c = 556.5, β = 92.4°
Wavelength (Å)	0.976	0.976
Dmin (Å)	3.0	3.5
Number of crystals	6	7
Number of images	80	101
Rotation per image (°)	0.3	0.3
Number of unique reflections	362,178	491,997
R merge(%)*	28.4	43.8
Completeness (to Dmin Å) (%)†	41.1 †	22.9
Average redundancy per shell (outer shell)	1.5 (1.3)	1.4 (1.0)
**Refinement**		
Data range (Å)	20–3	15–4
Number of unique reflections	327,266	403,441
R factor (%)‡	27.5	45.9
Protein atoms	6511	6682
Solvent	N/A	N/A
Non protein atoms	N/A	N/A
r.m.s.d. bond lengths (Å)§	0.018	0.018
r.m.s.d. angles (°)§	2.4	2.5
r.m.s.d. B main chain (Å2)	1.0	2.0
r.m.s.d. B side chain (Å2 )	1.0	1.0
Average B-factor (Å2)	7.0	11.0

*: R = 100×Σh Σj | |Ih, merged| - |Ih, j∥/Σh N|Ih, merged|, where j = 1, …, N for N data sets.

**†:** Completeness of the highest resolution shell 3.11–3.0 Å was 46%.

**‡:** R = 100×Σh | |Fh, obs| - |Fh,calc∥/Σh |Fh,obs|.

**§:** Root mean square deviation from ideal bond lengths or bond angles.

Since the pH of crystallization liquor for this structure was 4.6 (tests confirmed that the virus solution did not significantly perturb this) we might have expected to see dissociated pentamers rather then intact particles. However, the structure was clearly of an intact particle, although the disorder of certain features on the inside of the capsid prompted us to try to solve the structure of the virus at physiological pH. Crystals grown at neutral pH only yielded poor, incomplete data to 3.5 Å (see Materials and methods) but possessed 120-fold non-crystallographic symmetry. Averaging rendered the electron density clear, delineated differences on the inside of the capsid compared to the low pH structure, and confirmed that there were no major changes elsewhere in the particle (Figure 2B).

### Native virus structure

**Figure 3:**
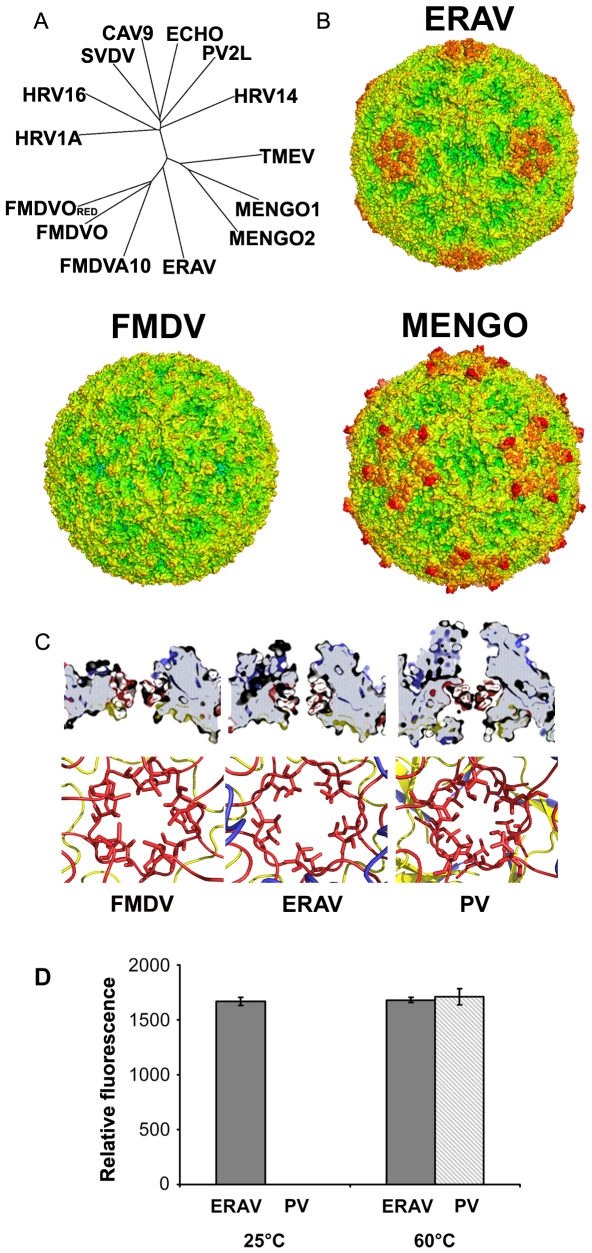
Structure comparison. (A) A phylogenetic tree [20] based on a structural alignment of complete protomers (VP1,2,3 & 4) for picornaviruses (RCSB protein data bank code (Berman et al. 2000)): Theiler's virus (1TME), Mengo virus 1 (1MEC), Mengo virus 2 (2MEV) FMDV A10 (1QBE), FMDV O (1BBT), FMDV reduced (1FOD), HRV 14 (4RHV), HRV 16 (1ND2), HRV 1A (1R1A), Swine vesicular disease virus (1OOP), Coxsackie virus 9 (1D4M), Echovirus 1 (1EV1), PV 2 (1EAH). (B) Surface depictions of FMDV (1FOD), ERAV and Mengovirus (2MEV) coloured by radial height (with the same colour scheme for all three particles) to illuminate surface features [55]. (C) 5-fold pores. FMDV, ERAV and PV 1 Mahoney (1HXS) are shown, above as slices through icosahedral 5-fold axes (the axes are vertical), below looking down the pore. In each set of representations the view-point and scale is the same for all three viruses. (D) Capsid porosity measured by the accessibility of viral RNA within the capsid to an RNA binding fluorescent dye at 25°C and after thermal uncoating by incubation at 60°C for ERAV and PV 1 Mahoney.

A structure–based phylogenetic tree constructed from similarities between the biological protomers of picornaviruses (Figure 3A, [20],[21]) indicates that ERAV is most similar to FMDV.

FMDV has short surface loops, so that the virus presents a rather smooth surface (Figure 3B) with an outer radius of 152 Å. In contrast the surface profile for ERAV extends to an outer radius of 159 Å at a prominent crown around the five-fold axis built from the extended VP1 loops (Mengo virus has an outer radius of 164 Å). ERAV also possesses marked surface depressions or pits around the five-fold axis, not dissimilar to those which harbour the site of receptor attachment in Mengovirus [22] (Figure 3B). The receptor-binding and antigenic properties of ERAV will be described elsewhere (Fry et al., in preparation).

FMDV capsids, unlike those of enteroviruses, contain pores penetrable by large molecules and ions such as proflavine and Cs^+^ [23]. The ERAV structure reveals the presence of capsid pores similar to those seen in FMDV, at the 5-fold and 3-fold axes of symmetry (Figure 3C). To confirm the porosity of ERAV, ERAV or PV was mixed at 25°C with a dye (ribogreen) which fluoresces upon binding to RNA (Figure 3D). With ERAV, a strong increase in fluorescence indicated penetration of the capsid by the dye; however with PV no increase in signal was detected, consistent with the PV capsid being impenetrable by the dye. After thermal uncoating at 60°C, the signal from PV became equivalent to that of ERAV. Properties of the dye such as molecular weight and hydrodynamic radius were withheld by the manufacturer.

#### VP1

**Figure 4:**
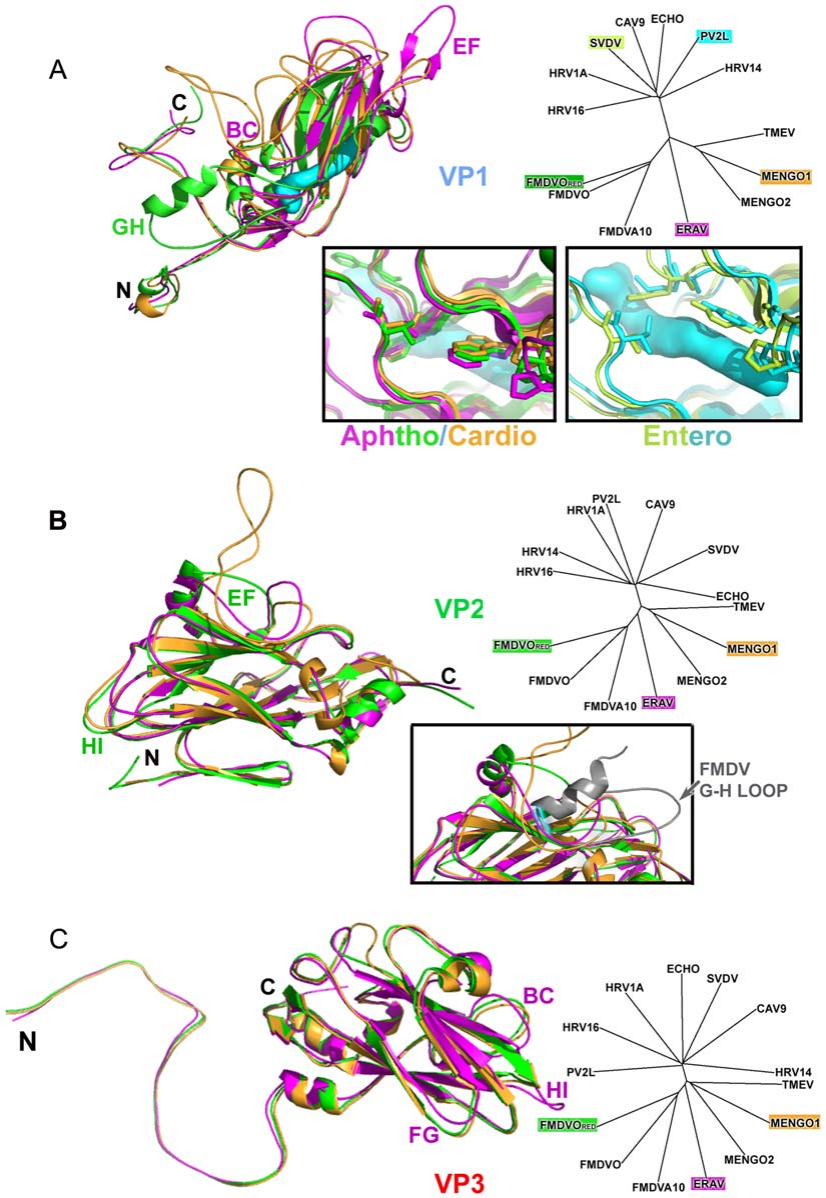
Protein comparison. (A) VP1: Overlay [55],[56] of ribbon depictions from ERAV (magenta), FMDV (1FOD) (green) and Mengovirus (2MEV) (orange) together with the structure derived phylogenetic tree (as Figure 3A) for VP1 proteins with those overlaid highlighted in the same colour. Inset are close-ups of the VP1 pocket in the aphtho and cardioviruses (left) and enteroviruses (poliovirus type 2 Lansing (1EAH) in cyan and, as a further example, swine vesicular disease virus (1OOP) in lime green, right). Both panels include a surface representation in cyan of a sphingosine pocket factor (as bound to swine vesicular disease), although since the site is occluded in aphtho and cardioviruses it is semi-transparent in the left-hand panel. (B) VP2: Overlay and phylogenetic tree (as [A]) for VP2 proteins. Inset is a close-up showing the closer similarity in the VP2 EF loop between ERAV and Mengovirus and the correspondence with the VP1 GH loop (shown in grey with the portion harbouring the integrin binding RGD motif highlighted with a cyan glow) in the reduced FMDV structure (1FOD, [28]). (C) VP3: Overlay and phylogenetic tree for VP3 proteins (viruses, representation and colours as for [A]).

The narrow end of the β-barrel packs around the five-fold axes of the icosahedral particle, defining the walls of the five-fold channel. Every residue (1–246) could be clearly identified in the averaged electron density map. Overall this protein deviates the most from FMDV - an extra 33 residues are disposed as insertions in the surface oriented loops: DE, HI, BC, EF and GH. Some of these extra residues match the longer Mengovirus VP1, so that ERAV VP1 is structurally equidistant from FMDV and Mengovirus (Table 2 & Figure 4A).

**Table 2:** Structure comparisons of ERAV with FMDV and Mengovirus.

Polypeptide	FMDV	Mengovirus
VP1	2.0 Å (186)	2.4 Å (213)
VP2	1.8 Å (204)	2.5 Å (217)
VP3	1.5 Å (217)	1.7 Å (221)

The FMDV and Mengovirus structures are 1FOD and 2MEV respectively [11] and the structure comparisons were performed using SHP [56]. The root mean square deviation between the overlaid structures is given with the numbers in parentheses, the number of Cα atoms matched.

The first fifteen N-terminal residues form a loop bridging between VP2 and VP3 on the inside of the particle within a protomer (as seen in other picornaviruses, although in enteroviruses the N-terminus is extended, so that these residues are analogous to residues 44–56 in poliovirus type 1 [24]. The BC loop (residues 40–54) (Figure 4A), an important antigenic site in other picornaviruses, has an extra nine residues in ERAV relative to FMDV. In this position there is a major insertion in Mengovirus (loops CD1 and CD2). A cis-proline observed at VP1 111 in FMDV is not conserved in ERAV. The EF loop (residues 110–140) is 18 residues longer in ERAV than FMDV and 8 residues longer than in Mengovirus (Figure 4A). The extended loop (a finger-like projection comprising a two-stranded anti-parallel β-sheet) initially follows the structure of the EF loop in Mengovirus but subsequently extends, wrapping around the five-fold axis (anticlockwise) in close proximity to β-strands B and I contributing an outer layer to the crown at the five-fold axis. The GH loop, which is immunodominant and highly flexible in FMDV, is 14 residues shorter in ERAV (Figure 4A) and quite similar to the equivalent loop in Mengovirus. In both FMDV and ERAV, the C-terminus of VP1 traverses the outer surface of the virus clockwise over a 5-fold related VP1subunit.

In the enteroviruses VP1 contains a hydrophobic pocket which in some cases is occupied by a fatty acid-like molecule or ‘pocket factor’ and may regulate viral uncoating. In cardioviruses, FMDVs and ERAV this void within the β-barrel is filled by large side-chains [23],[25] (Figure 4A).

#### VP2

VP2 (230 residues in length) is 12 residues longer in ERAV than FMDV but 26 residues shorter than in Mengovirus. Structural comparisons show that it is most similar to FMDV (Table 2 & Figure 4B). The first 11 residues are disordered. Residues 14–27 form a hairpin contributing to an extended β-sheet which stabilizes the pentamer interface. Between residues 38 and 47 in the internal βA_2_-βB loop, the conformation is more similar to Mengovirus than FMDV, which may reflect differences in the structure of the C-terminal portion of VP4, proximal in FMDV. Residues 41–43 comprise one of the two regions which vary between ERAV isolates [26]. A notable deviation from FMDV VP2 is an insertion of 11 residues in the EF loop in ERAV (residues 137–148), to form a structure resembling ‘puff A’ in Mengovirus, though lacking ‘puff B’ [27] (Figure 4B). In ERAV the VP2 EF loop contributes to filling in the canyon with the extra residues folding down to overlap the position of the FMDV VP1 GH loop [28]. Residues Trp 142, Ser 143 and Glu 144 of ERAV superimpose very closely with the integrin recognition motif of FMDV, Arg145, Gly146 and Asp147 respectively. The other significant change is a small deletion (3 residues compared to both FMDV and Mengovirus) in the HI loop (203–207) where it approaches the icosahedral 3-fold axis. Residue 84 is a cis-proline; conserved in all picornaviruses seen to date. The C-terminal four residues follow the path of those in FMDV (the Mengovirus C-terminus has a different conformation).

#### VP3

VP3, the most conserved of the structural proteins is five residues longer in ERAV than in either FMDV or Mengovirus. Structural comparisons show that it is most similar to FMDV (Table 2 & Figure 4B). One of the most significant differences is in the HI loop (193–201), which approaches the 3-fold axis and pentamer boundary. In ERAV it is 6 residues longer than FMDV or Mengovirus. This extended loop compensates for shortening of the VP2 HI loop, maintaining the calcium binding site on the axis. The FG loop (148–152) adjacent to the N-terminal loop of VP1 adopts a conformation distinct from FMDV and Mengovirus, folding away from the VP2/VP3 interface. The BC loop (64–73) at the pentamer interface and the GH loop (171–180) adjacent to the C-terminus of VP1 also differ from the conserved conformations seen in FMDV and Mengovirus.

#### VP4

Only residues 16 to 36 of the 80 residues are visible in the electron density and these are indistinguishable in conformation from FMDV. In all FMDV structures there is no clear density for the central portion of VP4 (residues ∼39–69) but the C-terminal portion including a helical stretch comprising residues 69–85 is usually visible, overlaying the N-terminal loop of VP1. In Mengovirus residues 13 to 70 are ordered. ERAV, like FMDV has no clear electron density for the N-terminal myristate.

### Low pH particle structure

**Figure 5:**
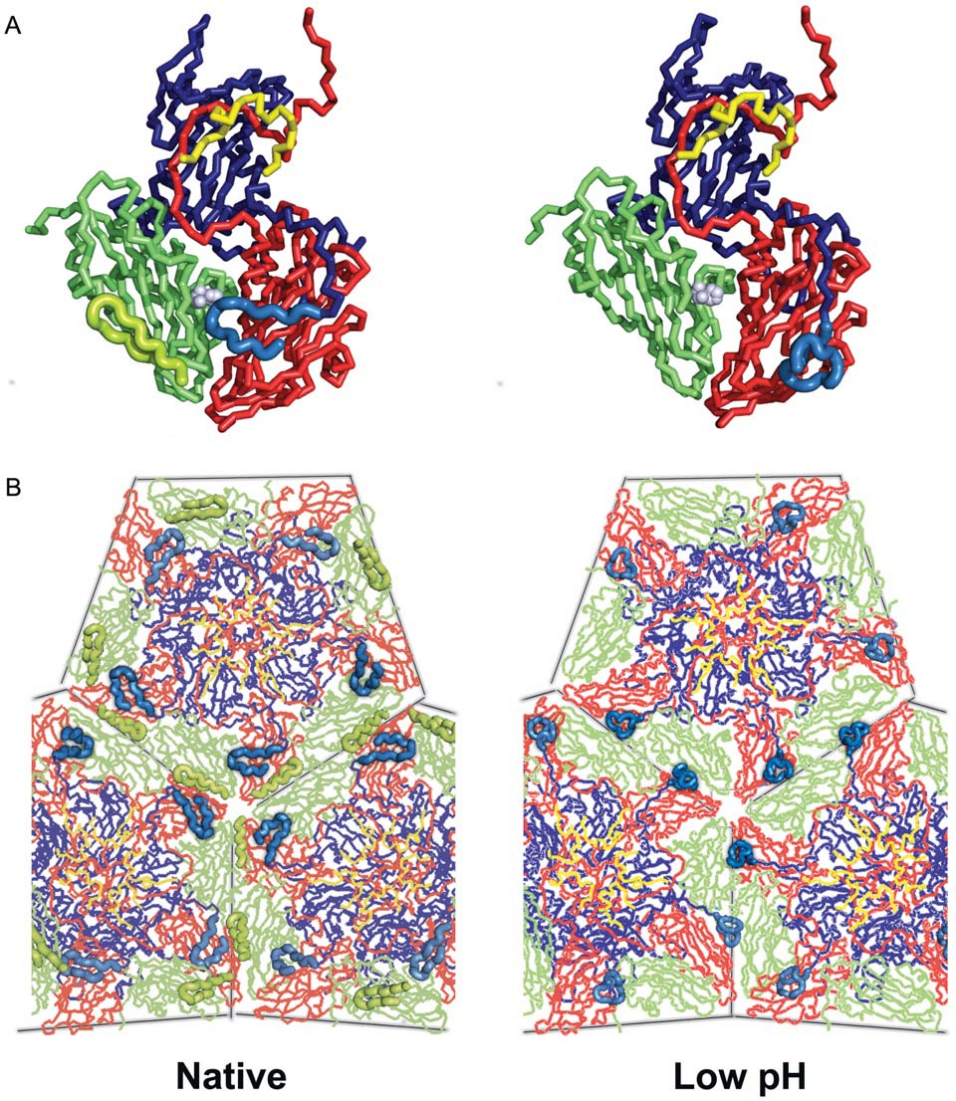
Proposed disassembly intermediate. (A) Tube depictions [55] coloured as in Figure 1A of the native (left) and low pH (right) protomer structures of ERAV, viewed from the inside of the particle. The regions which change between the structures are highlighted by using a thicker tube. In the native form the VP2 N-terminal hairpin (residues 12–30) is ordered and the N-terminus of VP1 adopts a loop structure stabilizing the VP2/VP3 interface. In the low pH form the VP2 hairpin is disordered and the N-terminus of VP1 has moved to the pentamer interface. His 160, the homologous residue postulated to be involved in the autocatalytic cleavage of VP0 is shown as grey spheres. (B) The variation in pentamer interactions. The representation is similar to (A) and the view is, again, from the inside of the particle.

The overall structure of the low pH particle is very similar to the native described above (for the protomeric unit 674 residues can be superimposed with rmsd 0.9 Å) *i.e.* there is no expansion of the low pH particle. There are however significant rearrangements, and an overall loss of order, in several internal loops. Thus the entire first 31 residues of VP2 cannot be clearly defined, including the hairpin structure which is generally well conserved and is a crucial element in stabilizing the pentamer interface. The VP1 N-terminus rearranges to form a loop close to the pentamer interface underlying VP3 and adjacent to the site of residues 78–88 of VP4 in FMDV (Figures 2 and 5). It thus occupies the position that would otherwise be occupied by the VP2 hairpin from an adjacent pentamer and replaces some of the stabilizing interactions at the pentamer interface, however overall the inter-pentamer interface between VP2 and VP3 is weakened. In contrast there do not appear to be any significant rearrangements on the capsid exterior surface, only slight deviations in loop conformations and side-chain orientations. The similarity between the two structures extends to the calcium ions bound on the icosahedral 3-fold axes (liganded by Asp 195 and Thr 194 of VP3) in both the low pH and native particles.

Allowing the electron density to float to different levels on the inside and outside of the capsid during cyclic averaging of the electron density map yielded essentially no difference between the inside and outside (this result was consistent when the analysis was done over the full resolution range and when restricted to data of 6 Å or lower resolution), whereas similar calculations for native FMDV particles showed a clear distinction with higher levels in the RNA rich interior than the solvent [29], suggesting that the low pH particle is empty. Unfortunately, since we were unable to measure data below 20 Å resolution, the reliability of this calculation is limited. However, empty particles which sedimented at 80S were detected following sucrose gradient centrifugation analysis of radiolabelled virus exposed to the low pH crystal buffer conditions (Figure S1).

## Discussion

### Structure of ERAV supports its classification

Structure based comparisons of the ERAV capsid with other picornaviruses (Figures 3 and 4) [20] reveal, as expected, a strong similarity to members of the aphtho and cardioviruses. For VP2 and VP3 it appears closest to the aphthoviruses but VP1 is intermediate between the two genera. Overall the structure supports the classification (based on sequence homology) of ERAV as an aphthovirus and the notion that it may prove a useful surrogate for studying FMDV biology without the need for high security bio-containment facilities.

### The picornavirus capsid and cell entry

Picornavirus capsids must provide a mechanism for the viral RNA genome to be safely transported from cell to cell. As stated in the introduction there are suggestions for how this may occur for enteroviruses, however, there is no such proposal for the cardio and aphthoviruses, which are thought to uncoat directly to pentamers.

### Structural basis for capsid disassociation

The seemingly distinct pathways for uncoating probably reflect the different architecture in the extended β-sheet which spans the pentamer interface in all picornaviruses. In aphtho and cardioviruses this comprises the VP3 CHEF strands from one pentamer and the VP2 N-terminal hairpin of the adjacent pentamer. In enteroviruses the extended VP1 N-termini contribute a further strand (residues 36–38 in poliovirus) so that strands from one pentamer sandwich the VP2 from the adjacent pentamer to stabilize the capsid. In FMDVs the protonation of a cluster of histidine residues (pI = 7.8) at the interface acts as a switch to dissociate the pentamers below pH 7, whilst ERAV, which in the native structure follows the canonical FMDV interface structure, has fewer histidines, consistent with the somewhat lower pH at which it dissociates into pentamers (between pH 6.5 and 5.5). In contrast the low pH ERAV structure shows weakened interactions at the pentamer interface, with no ordered electron density for the VP2 N-terminal hairpin, which is partly replaced by the N-terminus of VP1 from an adjacent pentamer which contributes a few stabilizing interactions (Figure 5B), consistent with a particle en-route to dissociation. This structure likely corresponds to the transient empty particle seen in the biochemical analyses but both experiments are suggestive of disassembly intermediates not previously reported. It is possible that the high particle concentration necessary for crystallization may prevent dissociation and/or drive the equilibrium towards the empty capsid intermediate.

### Capsid alterations leading to genome release

Enterovirus uncoating intermediates include the ‘altered’ 135S state [30],[31] and the 80S form devoid of RNA. Cryo-electron microscopy [31]–[33] of these particles revealed significant global alterations before and after genome release: externalization of myristoyl-VP4 and N-terminus of VP1, expansion of the particle, iris-like movement, opening of pores at 5-fold axes and movement of the VP2 ‘plug’. These structural transitions have led to models for mechanisms of membrane interaction, genome release and membrane penetration. In contrast the low pH ERAV structure remains relatively unaltered from the native capsid; the changes seen being restricted to internal features and reducing particle stability. No expansion of the particle is observed, there is no change in the diameter of the pores at the icosahedral 3- and 5-fold axes or in Ca^2+^ coordination on the 3-fold axes. The N-terminus of VP1 does not appear to be externalized. Interestingly the VP2 N-terminal hairpin is rich in hydrophobic residues and becomes disordered and may potentially be externalized and involved in membrane interactions.

In HRV2 and HRV14, pores at the 5-fold axes were seen to expand in the empty structures [32],[33]. However, for PV, pores large enough to allow passage of the RNA are not present in any of the structures and additional transient capsid forms have been proposed to explain genome release [34]. Similarly, there are no alterations in the ERAV pores between the low-pH and native structures to support their involvement in the exit of the genome. Additional RNA permeable intermediate structures must therefore also exist for ERAV. The formation of such structures may be facilitated by the reduced stability of the low pH particle.

The structure of a low pH form of the acid-dissociable Mengovirus [35] shows very few conformational changes compared to the native structure, with alterations confined to the receptor binding ‘pit’ consistent with a (pH dependent) loss of receptor binding prior to direct involvement with the membrane. This has been reported for HRV2 [36] and PV [16] and may be a generic feature of picornavirus cell entry.

### Role of VP4

In enteroviruses VP4 appears to play an important role in breaching the membrane. However, the low pH ERAV particle we have captured still contains ordered portions of VP4, although our results would be consistent with a number of copies of VP4 being lost from the particle. In this context we note that not all copies of VP4 are necessarily ejected from the 135S intermediate or empty particles of enteroviruses [33], [37]–[40].

### Low-pH rearrangements in ERAV – parallels to poliovirus

The final stage of picornavirus assembly is the cleavage of the precursor protein VP0 into VP2 and VP4. This is thought to establish a metastable state, priming the particle to initiate the entry process when receptor interactions and/or reduced pH trigger the conformational transition to a lower energy state. In poliovirus, interactions of the 44–56 loop of VP1 with the inner surface of VP2 and VP3 contribute significantly to the stability of the mature capsid and seem likely to have an important role in regulating structural transitions and cell entry. In a poliovirus immature empty capsid structure [41], where the cleavage of VP0 has not occurred, VP0 residues near the cleavage site prevent the N-terminus of VP1 from accessing its position in the mature particle. These final structural rearrangements to form the mature capsid involve similar structures to those externalized reversibly when the virus ‘breathes’ [42] and irreversibly in receptor-mediated conformational rearrangements early in the entry process [43]. The changes we see in ERAV correlate strongly with this (the residues rearranged in VP1 correspond to residues 44–56 in poliovirus), suggesting that cleavage and reorganization to prime the virus for the conformational changes required for cell entry [33],[34] is a general principle in all picornaviruses.

### Problems for uncoating are resolved by genome release from an intact particle

Our results suggest, surprisingly, that there are several common features between the uncoating process in aphthoviruses and the enteroviruses, including the existence of a quasi-stable 80S empty ERAV particle, produced upon acidification, which precedes disassociation into pentameric fragments. Furthermore, conditions have been previously reported which induce the formation of intact, empty FMDV particles missing both RNA and VP4 [44]. We infer from this that the ability to eject the genome while maintaining icosahedral integrity is a feature common to both aphtho and enteroviruses and suggest that there may be a general mechanism by which all picornaviruses protect their genome within intact capsids until the moment the genome is safely transported into the cytoplasm. Future research will more fully characterize these particles and the methods by which RNA is ejected.

## Materials and Methods

### Growth, sequencing and purification of virus

ERAV was provided by Dr Janet Daley (Animal Health Trust, Newmarket, UK). Sequencing confirmed the virus strain as NM11/67 (Genbank accession number FJ607143). Ohio HeLa cells were infected at low MOI and maintained at 37°C in DMEM (BioWhittaker) containing 5% FCS (GIBCO) and standard concentrations of glutamine and antibiotics. At complete CPE, cultures were freeze-thawed and pelleted by low speed centrifugation. Supernatants contained peak virus titres of ∼5×10^7^ pfu/ml (similar titres were obtained in Vero or RK-13 cells). The supernatant was precipitated by the addition of an equal volume of cold, saturated ammonium sulphate solution with mixing for 1 hour at 4°C. Precipitated material was pelleted at 10,000 g and 4°C for 30 minutes and resuspended in PBS, pH 7.4. Virus was purified by sedimentation through 15–45% sucrose gradients (w/v in PBS) by centrifugation at 111,000 g (average RCF, Sorvall AH629 at 29,000 rpm) and 4°C for 2.5 hours. The virus was located by measuring the 260 nm absorbance of gradient fractions. Peak fractions were pooled and precipitated with ammonium sulphate as before. Virus was re-purified by sedimentation through 15–30% sucrose gradients (w/v in PBS) at 237,000 g (average RCF, Sorvall AH650 at 50,000 rpm) and 4°C for 40 minutes. Peak fractions were pooled and samples concentrated and buffer exchanged for 50 mM Hepes pH 7.3, 50 mM NaCl, using a centrifugal concentrator (Vivascience). Poliovirus Mahoney strain (PV1) was propagated in HeLa S suspension culture and purified as previously described [16].

### Sucrose density gradient analysis of low pH induced products

Viruses with [^35^S]methionine and [^35^S]cysteine labelled capsid or [5,6-^3^H]uridine labelled RNA were generated by metabolic labelling of infected cells and purified as described above. Radiolabelled viruses were exposed at ∼20°C to differing pH values by diluting at least 20-fold into solutions containing 50 mM NaCl and 50 mM of either HEPES pH 7.3, MES pH 6.5, sodium citrate pH 5.5, or sodium citrate pH 4.5 (other conditions as mentioned in the text). Samples were layered onto 5 ml 10–30% sucrose gradients and centrifuged as described. Radioactive material was located by liquid scintillation counting (Packard Tri-Carb) and ^3^H and ^35^S signals distinguished by their energy spectra.

### Analysis of capsid porosity by dye penetration

Purified virus and ribogreen reagent (Invitrogen) were combined at room temperature at final concentrations of 10 µg/ml and 1 in 2000 dilution respectively and fluorescence (485/520 nm) measured at ∼25°C, using a BMG Optima plate reader. Thermal uncoating was performed by incubating samples at 60°C for ten minutes and cooling to 25°C before measuring the fluorescence.

### Crystallographic analyses

#### Low pH particle structure determination

The low yields of ERAV (typically 36 µg from 10,000 cm^2^ tissue culture), required a highly efficient crystallization strategy. Initial screens utilized Fluidigm TOPAZ chips [45] implemented at the Oxford Protein Production Facility. Five chips were set up using approximately 16 µl of virus at 3 mg/ml covering 2772 conditions (with varied protein concentration and temperature) plus buffer controls. Within an hour crystals were visible in numerous conditions, particularly with the Optimix 3 salt conditions [46]. A screen using the Cartesian dispensing robot [47] with 100 nl virus at a concentration of 2.7 mg/ml plus 100 nl reagent, yielded crystals of similar morphology to those obtained in the Fluidigm; principally thin rhombohedral plates from a variety of ammonium sulphate conditions and thicker crystals from 1 M di-ammonium hydrogen citrate, 0.1 M sodium acetate pH 4.6, the latter yielding promising 3 Å diffraction data. Optimization using the Cartesian and varying the pH, drop ratio and precipitant concentration, based around the di-ammonium hydrogen citrate condition, with a small quantity (37 µg) of virus [48], yielded sufficient crystals to support structure solution. Measurements on crystallisation drops confirmed that the pH was below 5. Crystals (average dimensions 100×50×10 µm^3^) were stabilized in 1.8 M di ammonium hydrogen citrate and exposed at room temperature in quartz capillary tubes at the ESRF, with useful data collected from station BM14, λ = 0.9796 Å equipped with a microdiffractometer [49] (100 µm diameter beam) and MAR 225 CCD detector. The clean beam, large detector and moderate flux allowed the collection of more data per crystal than at other available beamlines. Typically ten consecutive exposures with 0.3° oscillation were obtained per crystal showing diffraction to a maximum resolution of 3 Å. The crystals belong to space group *C222_1_* (a = 344.8, b = 531.4, c = 488.3 Å). The dataset, comprising 80 images, was processed using Denzo and Scalepack [50] and partially recorded reflections scaled up (D.I.S. unpublished program, statistics are given in Table 1).

The structure was solved by molecular replacement. Packing considerations dictate that the particle must occupy a two-fold axis (30-fold non-crystallographic redundancy). The self rotation function (maximum 5-fold peak 12σ), demonstrated that the orientation of the icosahedral particles exactly matched that of the PV2L particles crystallized in the same space group. A 30-mer search model was made from the FMDV A10 coordinates (1ZBE [11],[17]) rotated to the same frame and positioned with the particle centre at (0, 0, 0.25). A clear peak (7.5σ above the mean) in the translation search along y (XPLOR [51]) located the particle at y = 0.24. Rigid-body refinement (XPLOR [51]) and positional refinement yielded improved phase estimates (final R-factor to 3 Å 38.4%) and an electron-density map which showed clear changes relative to the starting model (Figure S2). Cyclic 30-fold non-crystallographic symmetry averaging (with 20–3 Å data) using GAP (D.I.S. and J.M. Grimes, unpublished), yielded an agreement between the observed data and those calculated from the averaged electron density map of R = 20.0%, CC = 0.88. The resulting map was extremely clear, enabling the correct ERAV sequence and structure to be built using O [52]. Rebuilding iterated with NCS constrained refinement [51] yielded the final model (Table 1).

#### Native virus structure determination

Crystals grown from 1.6 M ammonium sulphate, 0.1 M Hepes pH 7.0 were optimized by grid screens using the Cartesian, varying the drop ratio and precipitant concentration. The crystals (average dimensions ∼150×80×5 µm^3^) belong to space group *P2_1_* (a = 314.0, b = 497.8, c = 556.5 (Å), β = 92.35°) with two virions per asymmetric unit (120-fold NCS). These extremely fragile crystals were very difficult to manipulate and it proved more tractable to grow them in 0.5 mm quartz capillaries using a 3∶1 ratio of virus solution to precipitant [53]. Diffraction data were collected as for the low pH crystals. Up to 7 useful consecutive exposures of 0.3° oscillation were obtained per crystal to no better than 3.5 Å resolution. The dataset comprised 101 images. The extremely small diffraction volume, combined with the very large volume of the unit cell and high background scatter from the liquid surrounding the crystals rendered these data of poor quality (Table 1). Nevertheless the structure was solved by molecular replacement using the low pH ERAV model. Inspection of the self-rotation functions yielded the particle orientation and inspection of the native Patterson function followed by limited translation searches (XPLOR, [51]) positioned the particles at (0.13, 0.0, 0.38) and (0.38, 0.5, 0.13). Some peak-splitting was evident, but the extreme weakness of the data prevented this issue being resolved and this contributes to the poor quality indicators in the analysis. Rigid-body refinement (XPLOR, [51]) (30–10 Å data, CC = 0.59) yielded improved phase estimates which were used to generate an electron-density map. The map was refined by cyclic 120-fold non-crystallographic symmetry averaging (with 20–4 Å data) using GAP (D.S. and J. Grimes, unpublished). Upon convergence the agreement, using metrics defined above, was poor: R(%) = 43.8 (33.4) and CC = 0.53 (0.73) (figures in brackets correspond to (F>F_mean_)). Despite this the averaged 2|Fo|-|Fc| map clearly established the structural changes relative to the low pH ERAV structure and allowed a model to be built using COOT [54] and refined [51]. Atomic coordinates and structure factors have been deposited with the Protein Data Bank: accession codes: 2WFF (native) and 2WS9 (low pH).

## Supporting Information

Figure S1 Centrifugation analysis of radiolabelled virus. At pH 7.3 radiolabelled virus sediments at the expected rate of 150S following sucrose gradient centrifugation analysis whereas empty particles which sediment at 80S are detected for virus exposed to the low pH crystal buffer conditions.
(0.04 MB DOC)

Figure S2 Averaged initial map for the low pH particle. Electron density (yellow) obtained from simple averaging of the initial map for the low pH crystal form of ERAV. The map was obtained using 2Fo-Fc amplitude coefficients, where Fo were the experimental amplitudes for ERAV and Fc (and the phases for the map) were derived from the automatically refined molecular replacement model, FMDV A10 [17]. The final ERAV coordinates are shown in magenta and the A10 coordinates in cyan. Note that there are places where changes to the amino acid sequence from FMDV to ERAV are correctly indicated by the electron density.
(4.48 MB TIF)
