# Supplementary material for: Equine Rhinitis A Virus and Its Low pH Empty Particle: Clues Towards an Aphthovirus Entry Mechanism?
Source: PLoS Pathog. 2009 Oct 9;5(10):e1000620. doi: 10.1371/journal.ppat.1000620 (PMC2752993; doi:10.1371/journal.ppat.1000620)
Supplement: Figure S1 — Centrifugation analysis of radiolabelled virus. At pH 7.3 radiolabelled virus sediments at the expected rate of 150S following sucrose gradient centrifugation analysis whereas empty particles which sediment at 80S are detected for virus exposed to the low pH crystal buffer conditions. (0.04 MB DOC) [file ppat.1000620.s001.doc]

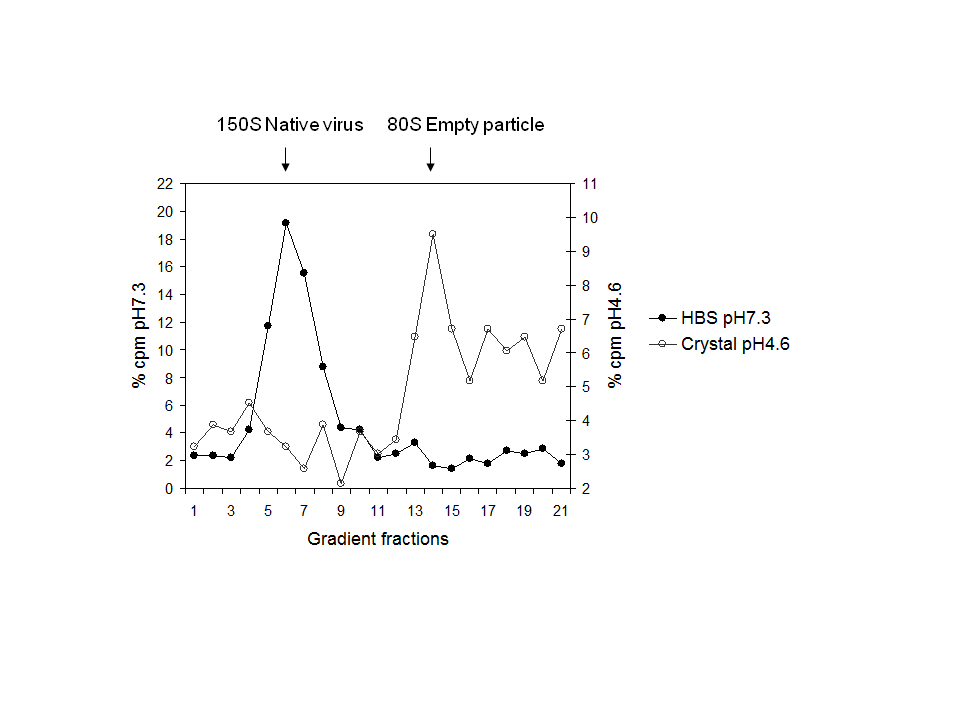


**Supporting Figure S1.**

**Centrifugation analysis of radiolabelled virus.**

At pH 7.3 radiolabelled virus sediments at the expected rate of 150S following sucrose gradient centrifugation analysis whereas empty particles which sediment at 80S are detected for virus exposed to the low pH crystal buffer conditions.
