# Supplementary figures and images for: Equine Rhinitis A Virus and Its Low pH Empty Particle: Clues Towards an Aphthovirus Entry Mechanism?
Source: PLoS Pathog. 2009 Oct 9;5(10):e1000620. doi: 10.1371/journal.ppat.1000620 (PMC2752993; doi:10.1371/journal.ppat.1000620)

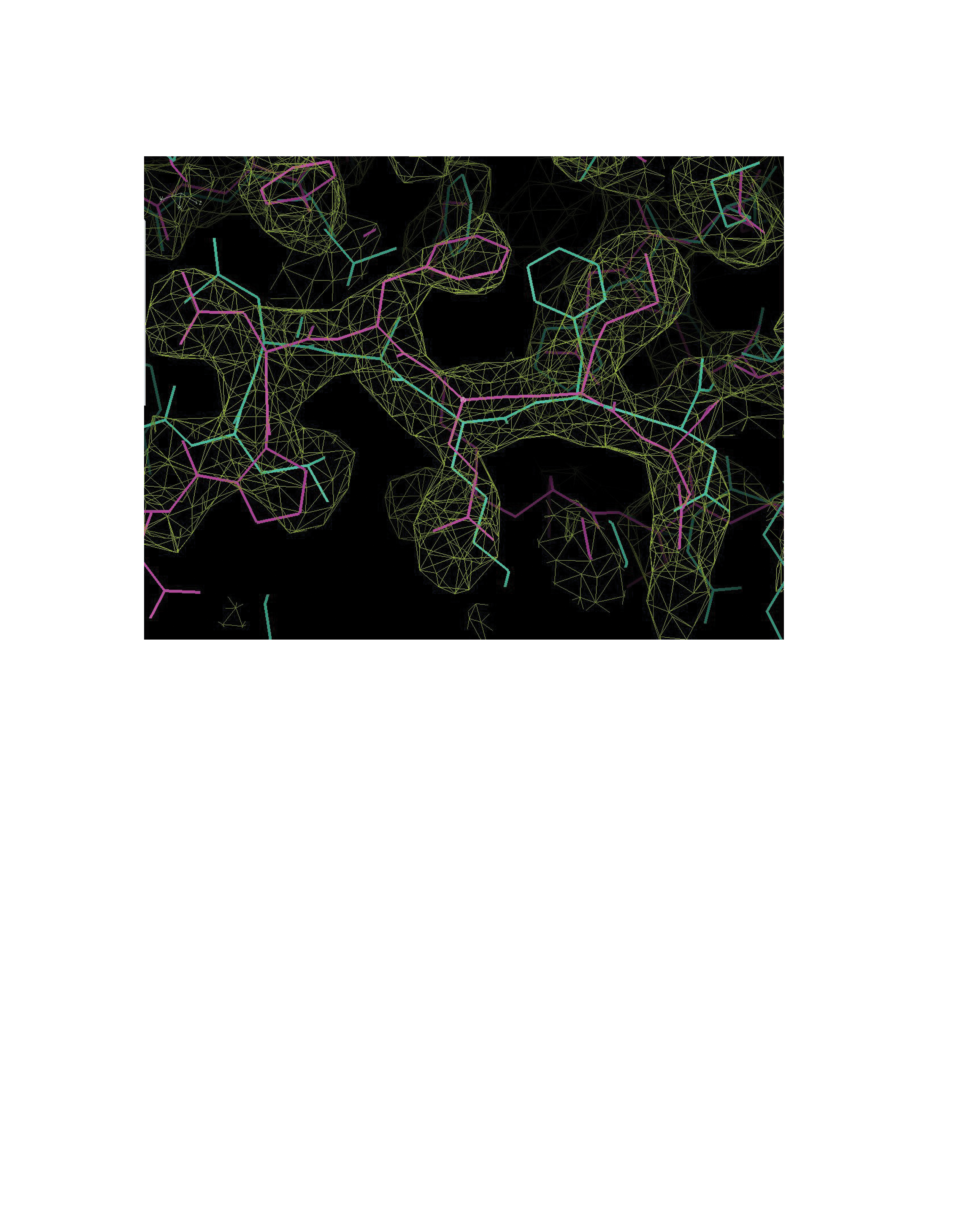

Supplement: Figure S2 — Averaged initial map for the low pH particle. Electron density (yellow) obtained from simple averaging of the initial map for the low pH crystal form of ERAV. The map was obtained using 2Fo-Fc amplitude coefficients, where Fo were the experimental amplitudes for ERAV and Fc (and the phases for the map) were derived from the automatically refined molecular replacement model, FMDV A10 [17]. The final ERAV coordinates are shown in magenta and the A10 coordinates in cyan. Note that there are places where changes to the amino acid sequence from FMDV to ERAV are correctly indicated by the electron density. (4.48 MB TIF) [file ppat.1000620.s002.tif]
